# Strategies employed in coping with physical disabilities acquired during adulthood in rural South Africa

**DOI:** 10.4102/ajod.v11i0.907

**Published:** 2022-08-05

**Authors:** Marubini C. Sadiki, Israel Kibirige

**Affiliations:** 1 Department of Research Administration and Development, University of Limpopo, Polokwane, South Africa; 2 Department of Mathematics, Science, and Technology Education, Faculty of Humanities, University of Limpopo, Polokwane, South Africa

**Keywords:** adulthood-acquired disability, coping strategies, support, experiences, rural community

## Abstract

**Background:**

Society places people with physical disabilities acquired during adulthood in disadvantaged positions, especially when they cannot participate in activities like their non-disabled counterparts. The situation can be worse for individuals who acquire disabilities during adulthood, where they have to learn to cope with the adulthood-acquired physical disabilities.

**Objectives:**

This study aimed to identify the types of physical disabilities acquired during adulthood and their causes and explore how participants defined their disabilities and the coping strategies they used.

**Methods:**

The study used a phenomenological research design. Five adults (three women, two men) with adulthood-acquired disabilities were purposefully selected from a rural area in Limpopo, South Africa. Data were collected using semi-structured interviews. Thematic analysis was used to generate themes about coping strategies study participants used.

**Results:**

The results show four types of adulthood-acquired disabilities amongst the participants: visual impairment, paraplegia, weakened muscles which led to bilateral amputation, loss of function on both hands and legs. Participants’ meanings of their physical adulthood-acquired disabilities ranged from a punishment, pain, not a bother, black magic, to results of doing wrong things to someone. In coming to terms with their adulthood-acquired disabilities, participants used problem- and emotion-focused strategies. Four themes from the participants’ responses were spiritual support, social support, substance dependency, access to health and rehabilitation services.

**Conclusion:**

The study contributes to understanding the experiences of individuals who acquired disabilities in adulthood, how they define their disabilities and the divergent coping strategies they use. This study established that participants used problem-focused, positive emotion-focused and negative emotion-focused coping strategies.

## Introduction

The World Report on acquired disabilities is an intricate and multifaceted concept because there are many definitions depending on the disciplines, such as medicine, sociology and politics (Mitra 2006). Historically, the concept of acquired disability was perceived as a religious myth, and various African cultures perceive it differently (Eskay et al. 2012). Acquired disability was seen as a punishment for wrongdoing, and such beliefs still exist in some societies (World Health Organization [WHO], UNESCO, International Labour Organization & IDDC 2010). As a result of these mixed perceptions, acquired disability has been associated with stigma, discrimination and isolation, which result in low self-esteem (Parr 2007). Whilst disabled persons have the same rights as anybody else, physical and social barriers in society limit their performance of specific tasks (WHO 2010). Many authors agree with the International Classification of Functioning (ICF) that disability of any form is a persistent condition interacting with individual, contextual and social factors (WHO 2001).

Adulthood-acquired disabilities (AADs) are disabilities that individuals gain after 18 years of age and after high school. Globally, 350 to 500 people acquire physical disabilities each day (Disabled World Tomorrow 2013). A few studies show that AAD may arise from illness or injury (Calderón-Larrañaga et al. 2018; Lisko et al. 2021; McAlpine 2008; Norman et al. 2007), and they come with significant changes to social, emotional and psychological well-being. The number of people with AAD globally is shocking. According to the Disabled Living Foundation (2021), 80% of all disabilities are acquired during adulthood between 18 and 64 years. People with AAD face discrimination challenges (Tagaki 2016) and social exclusion in societies (Lejzerowicz & Tomczyk 2018). Society applies an eugenic influence to consider persons with AAD as inferior in society (Watermeyer, McKenzie & Swartz 2019). Facing these circumstances, people with AAD develop low self-esteem (Bogart 2014) and may develop mood swings between positive and negative (Yoshida 1993). Disability is professed as problematic and needs treatment and rehabilitation (Buntinx & Schalock 2010). Thus, people with AAD need support to adjust to the acquired disability as a new way of life (Tagaki 2016; Yıldız & Cavkaytar 2020). People with ADD need social support to become resilient (Müller et al. 2012) and productive in society (MacLeod et al., 2016).

Rehabilitation is goal-oriented to enable people with AAD to attain holistic functioning (WHO 2011). In this study, rehabilitation is a means to expedite social adjustment to the new condition. Articles 20 and 26 of the United Nations Convention on the Rights of Disabled People (2006) address accessibility and rehabilitation, and it clearly states the measures to ensure that people with AAD access health and rehabilitation services (United Nations [UN] 2006). Rehabilitation depends primarily on the quality of the interactions between professionals working with disabled people and their families (Diken 2006; Pechak & Thompson 2007).

In Africa, 10% of the population lives with disabilities (WHO 2011). According to Munyi (2012), individuals with acquired disabilities are regarded as destitute, and they are regularly associated with everything evil (Lustig & Strauser 2007). In Zimbabwe, the most dangerous magician is a disability (Khupe 2010). In Namibia, each tribe approves traditional and cultural policies to guide families and communities regarding disabilities (Khupe 2010). For instance, a plate or cup used by a disabled person is considered ‘ritually unclean’, which can be good for feeding cats and dogs (Khupe 2010). Thus, these myths suggest that those with acquired disabilities are perceived to host evil spirits (Khupe 2010).

According to Statistics South Africa, the concept of physical disability has developed over a long time, and it is a physical or mental challenge lasting for 6 or more months and impeding a person’s physical functioning (Statistics South Africa 2014). Any impairment limits body functions, such as spina bifida, amputation, wounded spinal cord and dwarfism (Ross & Deverell 2010). Adults who acquire physical disabilities may experience activity limitations (Eide & Igstad 2013) and may need to adopt coping strategies to manage their new state of life. Coping refers to adjusting individuals’ behavioural and psychological efforts to accommodate internal and external stressors (Folkman & Lazarus 1984). It is the mental and behavioural effort devoted to overcoming stressful circumstances (Folkman & Moskowitz 2004). Coping strategies may be healthy or not (Folkman & Moskowitz 2004). Roth and Cohen (1986) highlighted two coping strategies, problem-focused and emotion-focused, later expounded upon by Moos (1992). Problem-focused coping strategies endeavour to change the distressed person’s conditions, whereas emotion-focused coping regulates the upsetting emotions (Chen et al. 2018; Folkman & Lazarus 1984). For example, Moos (1992) detailed (1) the problem-focused coping strategies to include four tenets: logical analysis (LA) measuring cognitive effort, positive appraisal (PA) involving accepting the reality of the problem, seeking support (SS) regarding information and support from others, and problem-solving action (PS), where a problem is directly addressed. (2) Emotion-focused coping strategies also encompass four tenets: cognitive avoidance (CA), where one avoids the reality of the situation; acceptance (A), where an individual accepts the conditions as they occur; seeking alternative rewards (ARs) suggest behavioural changes to engage in new satisfying activities; and emotional discharge (ED), where negative emotions are expelled. Positive emotion-focused coping involves consolidating links with others for emotional support. Negative emotion-focused individuals blame others, express hostility and panic about stressors (Chen et al. 2018). Despite the many models to explain the experiences of acquired disabilities, these models are not adequate to describe the lived experiences of people with AAD (Livneh & Martz 2012). To date, there are few studies regarding coping experiences of people with adulthood-acquired physical disabilities. Therefore, this study (1) identified the types of physical disabilities acquired by participants and their causes; (2) explored participants’ definitions of acquired physical disability and the strategies they employed in coping with adulthood-acquired physical disabilities.

## Methodology

### Research design

This research aimed at understanding strategies participants employed in coping with adulthood-acquired physical disabilities using a phenomenological research design (eds. Denzin & Lincoln 2005; Merriam 2009; Smith, Flowers & Larkin 2009). The phenomenological design was suitable because participants described their experiences of the disability phenomenon (Creswell 2012), thus revealing the quintessence of things (Lin 2013). The investigation was carried out in a rural real-life situation and no attempt was made to manipulate the phenomenon of interest (Kobus 2010). It employed ethical triage to avoid harm to participants, such as prioritising events as they occur and paying attention to the interviewees’ visual expressions (Buchanan & Warwick 2021). As a result of the sensitivity of the research topic, the author arranged counselling sessions for participants who required the service because of the potential of interviews resulting in psychological discomfort.

### Participants

Families with disabled adults in the Vhembe District in the Limpopo province were approached for participation, and the study sample was chosen based on having acquired the disability during their adulthood. Initially, seven participants were recruited using the linear snowballing technique, where the first participant was identified and referred the researcher to another person, and another person referred to another, up to the seventh participant. However, two participants withdrew before the end of the study, and they were not included in data analysis. The withdrawal was because of their relocation away from the study area. Creswell (2012) contended that a sample of 5–25 is good enough for a phenomenological study to yield rich data. Voluntary consent from the participants was obtained before data collection. In order to ensure the anonymity of participants, the researchers used codes (Participants A–E).

### Data collection instrument and procedures

Data were collected using semi-structured interviews and document analysis (Creswell 2012). Each participant was given an appointment that was convenient. All participants signed consent forms before interviews. An audio recorder was used to record the interviews, and each interview lasted between 50 and 60 min. This time was adequate to allow probing (De Vos et al. 2011). The responses were read back to the participants to ascertain that what was recorded reflected their views, thus prolonging the process to refine the findings. All interviews were conducted in Tshivenda. The Tshivenda version was translated to English, and the English version was back-translated to Tshivenda to ensure that meaning was not lost. An interview schedule with the following questions was used to obtain information from participants:

1. How and when did you acquire the disability?
2. How would you define disability?
3. What has been your experience of living with the disability?
4. What has helped you to cope with disability?

### Data analysis

Data from the audio-tape recorder were transcribed. The first author read and re-read the participants’ transcripts (Rubin & Babbie 2011). Data were thematically analysed using guidelines explained by Braun and Clarke (2006): reducing data, selecting significance from less significant issues, recognising vital points and constructing a framework to reveal real issues at hand. Thematic analysis was used to get the main points, notable topics and novel themes.

### Trustworthiness

The researchers enhanced credibility and trustworthiness by reading and re-reading the participants’ transcripts to confirm what was captured as true reflections of their views, and this process prolonged engagement (Lietz, Langer & Fruman 2006) and minimised bias (Lietz et al. 2006). After listening to the recorded data many times, the researcher transcribed and read the transcripts to capture lived experiences. The information was subsequently returned to the participants to confirm the interpretation and themes that reflected their concepts regarding strategies used to cope with adulthood-acquired physical disabilities.

## Findings

The results show that most participants had negative attitudes towards their disabilities. Four types of AAD amongst the participants were: (1) visual impairment, (2) paraplegia, (3) weakened muscles which led to bilateral amputation, (4) dysfunctional hands and legs. The types of participants’ AAD and their causes are shown in Table 1.

**TABLE 1:** Participants characteristics (*n* = 5).

Participant	Gender	Age (in years)	Type of disabilities	Causes and duration of the disability
A	Female	45	Visual impairment	Motor vehicle accident; eye injury, 1 year
B	Male	42	Paraplegic (loss of function in the lower part – participant using wheelchair)	Man fell from a roof he was repairing, 1 year
C	Female	42	Weakened muscles, both legs amputated (participant using artificial limbs and crutches)	Health complications arising from diabetes, 2 years.
D	Female	41	Loss of function in hands and legs (participant using wheelchair)	Motor vehicle accident, 3 years
E	Female	46	Both legs amputated (participant using wheelchair)	Motorcycle accident, 2 years

*Source:* Sadiki, M.C., Radzilani-Makatu, M. & Zikhali, M.P., 2018, ‘Acquired physical disability: Personal meanings in a rural South African setting’, *Journal of Psychology in Africa* 28(6), 514–517. https://doi.org/10.1080/14330237.2018.1547865

The prevailing types of disabilities amongst participants included visual impairment, and physical disability.

### Causes of disabilities

The participants gave brief accounts on how they had acquired the disabilities:

‘I acquired visual impairment when I was involved in a car accident. The injury affected my both eyes.’ (Participant A, Visual impaired, 25 November 2012)

‘I acquired physical disabilities when I slipped off the roof I was repairing. It is because of the fall that I lost function in the lower part.’ (Participant B, Paraplegic [Loss of function in the lower part – participant using wheel chair], 15 November 2012)

‘I acquired physical disability because of diabetes, where both legs were amputated.’ (Participant C, Weakened muscles in both legs amputated [Participant using artificial limbs and crutches], 03 November 2012)

‘I acquired physical disability when I was knocked down by a car whilst crossing a road and the driver failed to stop when the traffic light was red on his side. I am currently using a wheelchair.’ (Participant D, Loss of function in hands and legs [Participant using wheel chair], 03 November 2012)

‘My physical disability is because of a motorbike accident. I was under the influence of alcohol at the time of the accident. I collided head on with a truck. As a result, my both legs were amputated.’ (Participant E, Both legs amputated [Participant using wheel chair], 12 November 2012)

### Participants’ definition of disability

The participants’ meaning-making of their AAD ranged from associating it with punishment, pain, not a bother, to ‘black magic’. A few extracts from the participants are as follows:

‘To me, disability is a curse. I mean it is a punishment because I was not born like this, I was the only one injured during that accident.’ (Participant A, Visual impaired, 25 November 2012)

‘The disability is not a bother to me. I have learned to cope with it. I take it easy and learn to live like any other able-bodied people, which assist me to escape negative attitudes from my community.’ (Participant B, Paraplegic [Loss of function in the lower part - participant using wheel chair], 15 November 2012)

‘Disability is a result of doing wrong things to someone. Maybe it is a punishment or a payback. I do not know.’ (Participant C, Weakened muscles in both legs amputated [Participant using artificial limbs and crutches], 03 November 2012)

‘Disability is pain, which you cannot understand until you experienced. I feel I am a burden to my family.’ (Participant E, Both legs amputated [Participant using wheel chair], 12 November 2012)

‘Disability is “black magic”, meaning that possibly some members of my family bewitched me. I feel embarrassed whilst using this wheelchair. I strongly believe that this is a curse. You see, it is also a challenge to get information on health services. You have to rely on other people to tell you.’ (Participant D, Loss of function in hands and legs [Participant using wheel chair], 03 November 2012)

### Coping strategies

Four themes emerged regarding the coping strategies used by participants with AAD: (1) spiritual support, (2) social support, (3) substance dependency and (4) access to health and rehabilitation services. We elaborate on these themes here using verbatim statements of participants.

### Spiritual support

Participants found comfort in God as a source of support. The participants could not grieve alone because successful grieving interactions depend on significant human interactions and support from religious groups. Participants with religious beliefs coped better with their disabilities than participants with no religious affiliation. The following extracts from three out of five participants indicated that spiritual support helped them cope with their disabilities.

‘My brother in-law introduced me to the local ministry, which I attend every Wednesday, and I believe that things will change for me and that I will be independent.’ (Participant A, Visual impaired, 25 November 2012)

‘Many miracles are happening in that church and I hope God will also do miracles for me. I also do not miss any all-night prayer services.’ (Participant C, Weakened muscles in both legs amputated [Participant using artificial limbs and crutches], 03 November 2012)

‘The church played a significant role in my life. My pastor always says to me that he prays for me….’ (Participant D, Loss of function in hands and legs [Participant using wheel chair], 03 November 2012)

### Social support

Participants stated support groups encouraged them to interact by sharing their distressing experiences. The social interactions increased their self-esteem despite their immediate families and friends who did not fully understand their difficulties. For instance, three out of five participants made the following comments:

‘I began to realise that my situation was much better than that of others…There are others who are worse off … I am lucky that I can see although I cannot move without a wheelchair.’ (Participant B, Paraplegic [Loss of function in the lower part - participant using wheel chair], 15 November 2012)

‘Sharing my experiences with other disabled persons helped me to open up and get assistance from other physically disabled persons.’ (Participant A, Visual impaired, 25 November 2012)

‘I thought advocacy organisations could help me to link with other disabled persons who understand my plight better than persons with no disabilities.’ (Participant D, Loss of function in hands and legs [Participant using wheel chair], 03 November 2012)

### Substance dependency

Two participants explained that they resorted to alcohol and other drugs, such as ‘dagga’, to cope with their disabilities. They explained that these substances make them forget their disabilities. The following extracts from participants show how they used different substances to cope with physical disabilities:

‘When my two legs were amputated, I was frustrated. I made friends in the ward where I was admitted. When they smoked, they would ask me if I smoked and I would refuse. After a while, I joined them and started smoking dagga because I realised that I was getting bored and I was focusing on my disability. Many people have approached me to stop smoking … I do not see the importance of pleasing other people. I must please myself.’ (Participant B, Paraplegic [Loss of function in the lower part - participant using wheel chair], 15 November 2012)

Others indicated that they took overdose of medicines, hoping to alleviate the stress:

‘I do go to the hospital for check-up. I only go to see the doctor when I am sick. I take an overdose of painkillers so that I can keep sleeping. I get intimidated to talk with other people.’ (Participant A, Visual impaired, 25 November 2012)

### Access to health and rehabilitation services

Participants reported the critical role of health professionals in making sure that disabled people cope with acquired physical disabilities. For instance, four out of five participants made the following narrations:

‘Home visits by the social workers opened up my eyes. I did not know that physically disabled people can qualify for grants. I thought I had to be 60 years. Also, there are transport barriers to go to the rehabilitation centre because I use public transport, which does not have facilities to accommodate disabled persons.’ (Participant D, Loss of function in hands and legs [Participant using wheel chair], 03 November 2012)

‘I have learnt how to use a wheelchair. I was assisted by a physiotherapist. Without the wheelchair, I was crawling on my knees. I could not move from one place to another. The wheelchair makes my movement easy.’ (Participant E, Both legs amputated [Participant using wheel chair], 12 November 2012)

‘Occupational therapist gave me a wooden table, which I use for my meals. I was feeling helpless, discouraged and despondent, but now I see the light.’ (Participant C, Weakened muscles in both legs amputated [Participant using artificial limbs and crutches], 03 November 2012)

‘The one-to-one sessions with psychologists and social workers improved my life. The support I got gave me some hope and I understood disability in a different way.’ (Participant B, Paraplegic [Loss of function in the lower part - participant using wheel chair], 15 November 2012)

## Discussion

The study aimed to identify the participants’ types of physical disabilities, identify the causes of such acquired disabilities and establish how participants defined and coped with their AAD. Accidents and disease (diabetes) caused participants’ physical disabilities. It could be because accidents are part of our everyday life. For example, participants use cars, motorcycles, bicycles and work on construction sites with various types of machines that can harm the body if one does not use them with care.

The study participants defined their disabilities differently: as a curse, pain, punishment and black magic. The divergent definitions of disability are not surprising, because literature has reported that disabled persons have different views of their disabilities (Papadimitriou 2008; Saltes 2012; Tagaki 2016). Many disabled people depict their acquired disabilities negatively, as Loja et al. (2013) and Strømsø (2008) argued. The negative self-image of their disabilities may emanate from high levels of anxiety and depression (Mushtaq & Akhouri 2016; VanSwearingen et al. 1998) as they internalised their disabilities. Related to their definitions of their situations, participants indicated different ways they used to cope with disability. Four themes regarding participants responses are grouped into three coping strategies: (1) problem-focused (Moos 1992), (2) positive emotion-focused and (3) positive and negative emotion-focused coping strategies (Chen et al. 2018).

Only one participant (B) employed problem-focused coping strategies and could work as if he was not disabled whilst the rest (Participants A, C, D, & E) employed emotion-focused coping by resigning from the reality of their disabilities. The participants’ responses indicate that they employed positive and negative coping strategies. Participants sought support from different sources, such as religious and social remedies. Some participants had not yet come to terms with their newly acquired disabilities. They had hope in religious remedies and sought the clergy’s special prayers and counselling and believed in God’s healing power. Their belief in God was a source of hope and strength. To most participants, religion provided meaning in their lives, especially because many people’s understanding of disability is linked to religious beliefs (Diken 2006). Some participants indicated that religious institutions helped them to cope with their circumstances. Thus, attending religious meetings was a source of spiritual and psychological enrichment (Pelentsov, Laws & Esterman 2015). A few participants hoped for restoration to their original states through prayers and they drew their hope from the miracles reported taking place in the different churches. The use of religious metaphors in dealing with chronic conditions has been previously reported (Stein, Lewin & Fairall 2007).

### Problem-focused coping strategy

Participant B had a positive view of his acquired disability and used a problem-focused strategy to tackle the alternative lifestyle regarding the disability. He exhibited a positive, focused coping strategy, shown in his seeking help from health practitioners and rehabilitation to deal with his disability. He, however, indulged in substances. This observation agrees with Moos (1992) because participant B sought solutions regarding the acquired disability, such as spiritual, social and health help. This strategy is not surprising because Sheldon, Renwick and Yoshida (2011) suggested that adults who acquire disabilities look for solutions to their challenges as disabled persons. Participant B declared that he was lucky to be alive and was positive about his disability because he used his logic to appreciate that being disabled and alive was better than being dead. The positive sentiments observed here agree with Moos (1992), who contended that problem-focused individuals use LA, are positive in accepting reality and look for actions to face the disability. Hence, Participant B was positive about living normally and acting within the confines of his disability. The positive attitudes can be understood because he was mature, aged 42 years old. This observation parallels Chen et al. (2018), who found that age contributes to choosing problem-focused coping strategies in managing the disability, unlike teenagers. Thus, they seek PS to address the problem at hand.

### Positive emotion-focused coping strategy

The results show that Participants C, D and E showed negative sentiments about their disabilities but sought solutions to manage their emotions concerning their AAD. According to Chen et al. (2018), these participants used a positive emotion-focused coping strategy. The participants invoked spiritual, organisational advocacy and occupational therapy in this strategy. These coping strategies should minimise the emotions caused by their disability. Moos (1992) contended that emotion-focused strategies encompass CA, where individuals avoid the reality of their disability, seek alternative temporal solutions and engage in temporal activities to dismiss reality. Positive emotion-focused individuals improve their physiology (Kok et al. 2013) and emotional well-being (Fredrickson & Joiner 2002).

### Positive and negative emotion-focused coping strategy

The results show that Participant A was negative about the acquired disability. Judging from sharing her disability experiences with other people to get some guidance and attending gospel ministry programmes every Wednesday, Participant A used positive emotions. This observation agrees with Chen et al. (2018), who contended that someone looking for help from communities or friends is geared to using positive emotion-focused coping strategies. Conversely, the use of drug overdose to keep her sleeping to avoid the emotions is used to categorise her as using negative emotion coping strategies. This categorisation agrees with Chen et al. (2018), who asserted that individuals who avoid the reality of the problem are likely to use negative emotion-focused coping strategies. Participant A used drugs to mask the disability challenges. This behaviour is common and several studies have reported an association between disability and stress (Chen et al. 2018; Mushtaq & Akhouri 2016; Terrill & Molton 2018; Wongwan 2021).

Chetty (2011) reported that some people indulge in substances to seek temporary relief. Also, given the circumstances, confiding in peers with positive attitudes could assist the substance abusers (Ibrahim & Kumar 2009). Moos (1992) contended that emotion-focused people use CA, seek alternative rewarding activities, which do not solve the problem and expel the reality of the problem.

From the study findings, persons with disabilities found comfort in social relationships, families and other support groups who tried to understand their AAD. The disabled person’s immediate family members have a social responsibility to accept the disability and work with them day by day. In addition, associating with other disabled persons made them feel comfortable, as they shared experiences with people who could understand their disabled situations better than persons without disabilities (Sadiki, Radzilani–Makatu & Zikhali 2015). Thus, social support improves well-being among people with AAD (Elliott, Kurylo & Rivera et al. 2002), and this support is effective if provided for a long time (Devereux et al. 2015). The problem-focused individuals used institutional support from health professionals to cope with their disabilities. Thus, institutional support was used as part of the problem-focused coping strategies (Roth & Cohen 1986). The emotion-focused participants exhibited both positive and negative strategies to assuage the challenges caused by their disabilities.

### Contribution to the body of knowledge

The study contributes to understanding the experiences of people living with adulthood-acquired physical disabilities and the coping strategies to deal with their acquired situations. It is an inspiration to all humanity, which is at a potential danger of acquiring physical disabilities, to hope that life can be worth living after acquiring physical disabilities. The study established that participants used problem-focused and positive/negative emotion-focused strategies to cope with their disabilities. Notwithstanding the strategies used, there were challenges of access to healthcare, which is a right of every person with acquired disabilities (Heapa, Lorenzo & Thomas 2009; Mégret, 2008). Other barriers include the lack of information on health services, long distances to health facilities and poor public transport that did not favour disabled persons (Beatty et al. 2003; Harris et al. 2011; Maart et al. 2007). Hence, there is a need to sensitise the community to sympathise and assist disabled persons for a worthwhile living.

## Limitations of this study

This study explored the coping strategies of individuals with AAD. The snowballing method used to select the sample may have missed other disabled persons in the same area who could have provided additional valuable information. The study was conducted with a small sample drawn from one rural community in South Africa, which may not represent the country’s varied cultural and religious diversity. The results, therefore, cannot be generalised to other contexts and settings. The study was qualitative, involving few participants and a quantitative study would be needed to explore a large population in the country. In addition, a study to explore the coping strategies of family members taking care of disabled adults may be needed.

## Conclusion

The study identified four categories of AAD: visual impairment, paraplegia, weakened muscles which led to bilateral amputation, dysfunctional hands, and legs. Most participants exhibited negative sentiments towards disabilities and only one was positive. The study established that participants used different coping strategies to deal with their disabilities and participants used different support services to manage or minimise the stress associated with their disabilities.
